# An insight into bisphenol A, food exposure and its adverse effects on health: A review

**DOI:** 10.3389/fnut.2022.1047827

**Published:** 2022-11-03

**Authors:** Muhammad Faisal Manzoor, Tayyaba Tariq, Birjees Fatima, Amna Sahar, Farwa Tariq, Seemal Munir, Sipper Khan, Muhammad Modassar Ali Nawaz Ranjha, Aysha Sameen, Xin-An Zeng, Salam A. Ibrahim

**Affiliations:** ^1^Guangdong Provincial Key Laboratory of Intelligent Food Manufacturing, Foshan University, Foshan, China; ^2^School of Food Science and Engineering, South China University of Technology, Guangzhou, China; ^3^National Institute of Food Science and Technology, University of Agriculture, Faisalabad, Punjab, Pakistan; ^4^Department of Food Engineering, University of Agriculture, Faisalabad, Punjab, Pakistan; ^5^School of Food and Agricultural Sciences, University of Management and Technology, Lahore, Pakistan; ^6^Institute of Food Science and Nutrition, University of Sargodha, Sargodha, Pakistan; ^7^Department of Food Science and Technology, Government College Women University Faisalabad, Faisalabad, Pakistan; ^8^Food Microbiology and Biotechnology Laboratory, North Carolina Agricultural and Technical State University, Greensboro, NC, United States

**Keywords:** bisphenol A, endocrine disruptor, synthetic chemical, polycarbonate plastics, epoxy resins, BPA toxicity

## Abstract

Bisphenol A (BPA) is a synthetic chemical widely employed to synthesize epoxy resins, polymer materials, and polycarbonate plastics. BPA is abundant in the environment, i.e., in food containers, water bottles, thermal papers, toys, medical devices, etc., and is incorporated into soil/water through leaching. Being a potent endocrine disrupter, and has the potential to alter several body mechanisms. Studies confirmed its anti-androgen action and estrogen-like effects, which impart many negative health impacts, especially on the immune system, neuroendocrine process, and reproductive mechanism. Moreover, it can also induce mutagenesis and carcinogenesis, as per recent scientific research. This review focuses on BPA’s presence and concentrations in different environments, food sources and the basic mechanisms of BPA-induced toxicity and health disruptions. It is a unique review of its type because it focuses on the association of cancer, hormonal disruption, immunosuppression, and infertility with BPA. These issues are widespread today, and BPA significantly contributes to their incidence because of its wide usage in daily life utensils and other accessories. The review also discusses researched-based measures to cope with the toxic chemical.

## Introduction

Xenoestrogens or endocrine disruptors are natural or synthetic compounds harmful to the endocrine system because they stop endogenous hormone production and normal functioning (1). Due to rapid advancement in human lifestyle, endocrine-disrupting chemicals are being introduced competently into the environment extensively, and living beings are directly or indirectly exposed to harmful chemicals such as Bisphenol A (BPA) (2–4).

Bisphenol A is among toxic chemicals, first highlighted by Aleksandr Dianin in 1891, then in 1905, they were made by Zincke using acetone condensation with two correspondents of phenol. In the mid-twentieth century (1940), a sudden rise in polymers (polycarbonates, polysulfone, polyacrylate, and epoxy resins) was observed with BPA. The polymers were also used as an antioxidant and endpoint for inhibiting polymerization in polyvinyl chloride plastics. Besides, flame retardant polymers, including tetrabromobisphenol-A were also prepared using the polymers (5). It makes different polymers, such as epoxy resins, polycarbonates, and other polymer materials (6, 7). Epoxy resins and polycarbonates were in high demand in 2015, 64 and 34%, respectively. Demand increases are expected with each passing year (8).

Additionally, in recent years, their uses have expanded to produce optical and electronic materials. Polymers also produce plastic food containers, drinking glasses, bowls, cups, and microwave-safe utensils (9, 10). Canned materials can be a significant source for food adulteration owing to direct contact since epoxy resins are utilized to protect the can from the inside (11, 12). They are used in other industries like the ink and paint industry, manufacturing of thermal papers, compact discs, electronics etc. (12, 13).

Since then, BPA has been abundantly used in food packaging materials, take-away water bottles, and lacquer coatings for tin cans causing human exposure to BPA *via* food and drinks (2–4). Furthermore, occupational workers get BPA exposure through direct contact with skin or inhalation, whereas the standard population is exposed to BPA by dust inhalation (14, 15). BPA has been linked to several serious health issues in animal model research. Numerous human-based epidemiological and observational studies on BPA exposure revealed similar results. BPA exposure is associated with the incidence of growth disruption, halting normal development, infertility, endocrine system disruption, immune system suppression, and carcinogenicity (16, 17).

By keeping in view the scenario mentioned above of BPA production and its utilization in different domains of life, the present review is aimed to elaborate on the different human BPA exposure routes and adverse health effects of toxicity with a special focus on basic mechanisms of endocrine disruption, infertility, carcinogenesis, and immunosuppression.

### Global production

The estimated global volume of BPA utilization was 7.69 million metric tons in 2015 for different applications that were forecasted to increase to 7.7 million in 2016. Approximately 4.8% compound annual growth rate (CAGR) is observed from 2016 to 2022. The production is predicted to increase to 10.7 million metric tons in 2020 because of the broad applications of polycarbonate plastics and epoxy resins in every field. The global demand for BPA is estimated to reach USD 22.49 billion in 2022. It was recorded to be approximately USD 15.6 billion in 2015, with a forecast of 16.4 as of 2016, marking a faster CAGR of 5.4% in value. The largest market of BPA is located in the Asia Pacific, contributing to approximately 52% of the market share, while 36% is produced by the USA and Western Europe (18, 19). Due to these applications, its extensive scale application is observed in everyday life, such as in producing papers, toys, water pipes, electronic products, and other plastic materials (20, 21).

### Physico-chemical properties

The molecular weight of BPA [4, 4-isopropylidenediphenol; 2, 2-bis (4-hydroxyphenyl)-propane] is 228.29 g/cm^3^, with a white crystal-like appearance and highly reactive due to the presence of hydroxyl group in the structure. The melting and boiling points of the toxic chemical are 156 and 220°C (at 5 hPa), respectively. The coefficient of BPA in water octanol is expressed in a logarithmic form value of 3.32 (log *P* = 3.32), indicating its high solubility in fats and less soluble in water (about 200 mg/dm^3^ at 25°C). Moreover, it can also be transformed into the ether, esters, and salts like other phenols. Additionally, the electrophilic substitution of BPA generally includes sulphonation, alkylation, and nitration (20, 22).

### Applications of bisphenol A

Bisphenol A is a well-known synthetic chemical globally used to manufacture different polymers, including epoxy resins, polycarbonates, and other polymer materials. Polycarbonates and epoxy resins are prominent polymers in significant bisphenol applications. Some other uses of bisphenol A include the production of different resins (unsaturated polyester, polysulfone, polyetherimide, and polyacrylate) (6, 7). In 2015, global demand for polycarbonates and epoxy resins was 64 and 34%, respectively. Moreover, the rise in demand for these two polymers will be observed with an average annual rate of 3 and 4% for the next 5 years (8). Furthermore, in recent years, their applications extended to manufacturing optical and electronic materials. Plastic cups, bottles, bowls, food containers, and utensils used for microwaves are also synthesized with polymers (9, 10).

Epoxy resins protect the can from the inside; therefore, they can be a considerable source for the adulteration of food items due to direct contact (11, 12). The storage bottles are also layered with epoxy resins for a similar purpose (23). Nonetheless, epoxy resins are also successfully applied in the paint and ink industry. Beyond this, epoxy resins also have a well-established reputation in manufacturing thermal paper, compact discs (CD), and digital video discs (12, 13). Whereas the derivate compounds from BPA are used in tickets and newspapers for antioxidants and stabilizers (15, 24) and, in the textiles industry, it is employed for infant socks preparation (25).

### Exposure to bisphenol A

Bisphenol A is present almost everywhere in our surroundings and significantly affects our life. It can be part of the food and environment directly or indirectly, affecting living organisms.

#### Environment

Bisphenol A is an ‘omnipresent’ contaminant due to its presence in all possible resources that might be the source of its human exposure through air, water, and soil (26). There are three main routes for human exposure environmental, occupational, and contaminated food consumption (27). Workers synthesizing BPA and their related derivative compounds (i.e., polycarbonate, epoxy resins, and polyvinyl chloride) are easy targets for occupational exposure. The main reason for the environmental exposure is the contamination of the atmosphere, soil, and aquatic systems owing to the BPA entering the environment due to its use in thermal paper recycling and relevant industries (27, 28).

According to the findings of Zhang et al. (29), who assessed the BPA concentration in water of different areas of China, 19 out of 20 water treatment plants had 5–14 ng/L of BPA. A similar situation was reported in Canada, France and South Germany. The main reason behind the increased incidence of BPA is the increased occurrence of epoxy resins and polycarbonate plastics. According to the Global Bisphenol A (BPA) Market Report and Forecast 2021–2026 report, the worldwide BPA market was $10.92 billion in 2020. The expected Compound Annual Growth Rate (CAGR) is 7.8% between 2021 and 2026 (30). Manufacturing of BPA products, their utilization, aging, and disposal in the environment are the major reasons behind the addition of BPA in ecosystems (31). Point sources include effluents from sewage treatment facilities and landfill leachate, whereas non-point sources include epoxy resin and polycarbonate plastic shards that infiltrate aquatic bodies (32).

According to various studies, approximately 56 μg/L of BPA can be ingested from the aquatic environment, 1–150 μg/kg from soil (33, 34), while 2–208 ng/m^3^ of BPA can be inhaled from the surroundings and dust contributes 0.2–17.6 μg/g contamination (35). In addition, contaminated seafood ingestion, metallic food cans, and plastic bottles can contribute 13.3–213 μg/kg, 2–82 ng/g, and 0.234 μg/L, respectively (36), whereas landfill leachates (17.2 mg/L) (37), dermal route (7.1–71 μg/day) (38), and dental material (0.013–30 mg/day) also contaminate the environment (39).

#### Food

Food exposure is the most important because fulfilling daily dietary needs is essential for survival (Table 1). Contamination of BPA through food exposure occurs due to the use of BPA for manufacturing different types of plastic containers [polycarbonate (PC) and polyvinylchloride (PVC) plastics] used for food serving and exposing their direct interaction with food. Epoxy resins are also used to manufacture food cans for inner coatings. Therefore, canned food products also play a significant role in adulterating food items. Residual monomers of these compounds migrate from the can to the food product, and food consumption causes safety issues in individuals (11, 12). Besides, food packaging materials are the primary cause of BPA accumulation in human beings. It is due to the penetration of BPA from packaging into foodstuff and beverages (40, 41). There are also secondary reasons which lead to exposure to BPA and hence the infected human population (25, 42, 43).

**TABLE 1:** Level of BPA (g/kg) in different food commodities.

Food	BPA level	References
Cereals	0.9–3.7	(118)
Fish	7.2–103	(119)
Fruits and vegetables	10.99–94	(120)
Canned (fruits and vegetables)	3.6–267	(121)
Canned soft drinks	0.033–3.9	(122)
Milk	1.33–175	(123)

##### Migration of bisphenol A particles into the food system

The migration of particles from the wrapping material to the food material is quite a complex phenomenon. It depends on different factors, including the composition of different food items, duration of contact time, the food temperature during contact, and packaging material type refs. Studies revealed that fat in foods also contributes to the migration of particles. Similarly, there is also a direct relationship between the square root of contact time and the concentration of molecules being migrated. Moreover, the high-temperature also leads to a rapid migration rate of residues (11, 12). BPA penetration into packaged products accelerates at higher temperatures (used for boiling water) than the lower temperature, around 20°C. It is also illustrated that the migration rate could be 55 folds more than the latter temperature (44). Food present in packaged products shows less absorption of BPA than the food preserved in canned materials with a standard concentration of 0.45 ng per 100 g (45).

Moreover, compounds like epoxy resins and PVC are used in manufacturing industries to protect the inner side of the can from corrosion and rust development due to direct contact with different food items (23). Bottles used for storage purposes also have such types of protective glaze. Monomers residues of BPA migrate into the food during high-temperature processing and storage in these bottles due to incomplete polymerization (23, 46).

Babies fed on mother milk or non-PC bottles had minimum BPA levels compared to babies using PC-free packaging. BPA levels were low compared to infants’ body mass using non-PC feeders and eating solid foods (6–36 months) (47). The research was conducted in two steps to check BPA levels in fresh, frozen, and canned foodstuff (using 204 samples). Firstly, they checked the BPA concentrations in canned products and then calculated BPA intake through diet. Results showed that the foods not packed in canned material had lower BPA levels (7%) than canned foods (73%), while dietary assessment of adults revealed canned coatings materials as the main BPA contributor. Altogether 12.6 ng/kg was calculated per day in the human body, out of which 12.4 ng/kg was penetrated from canned foods. Moreover, 3–12.95 ng/kg per day was the tendency of dietary consumption. In contrast, 30–70 ng/kg/day was determined in the urinary bladder, higher than the central capacity of dietary intake (48).

#### Human exposure to bisphenol A

**FIGURE 1:**
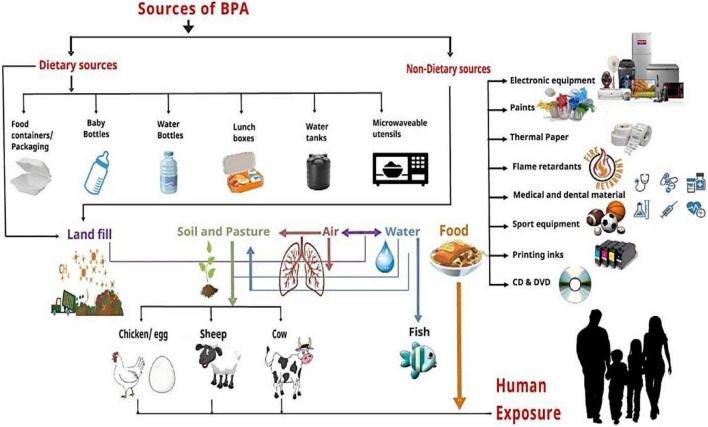
Humans exposure to BPA *via* different sources and exposed roots. Dietary sources (food containers/packaging, baby bottles, water bottles, lunch boxes, water tanks, and microwave utensils) and non-dietary sources (electronic equipment, paints, thermal paper, flame retardants, medical and dental materials, sports equipment, printing inks, and DVDs) contaminate landfill, soil, air, water, and food that directly or indirectly affect the human through different exposure routes.

Bisphenol A is almost everywhere in our environment and is released from common consumer goods (Figure 1). It may enter our body through different routes like dermal and oral exposure or only through inhalation (49). The primary route of exposure is dietary exposure, including consumption of seafood or even freshwater fish polluted *via* BPA, fresh food commodities from polluted regions, ingestion of food packed in plastic and cans containers, and drinking polluted water (50). The second foremost route of absorption for BPA is dermal exposure (51). Direct paper contact (especially thermal paper), toys, and medical devices proportionally increase the BPA potential against the skin. Inhalation is the third most important route of exposure through BPA-containing vapors, mists, dust, and gases (50).

Bisphenol A -human exposure’s primary source is canned food items. Therefore, this compound’s exposure mainly depends on the duration and amount of canned food usage in a person’s diet regimen. In kids older than 3 years, BPA exposure’s highest mean value was approximately 69.9 ng/kg body weight/day, with an utmost value of 189 ng/kg body weight/day. In adults, 139.9 ng/kg body weight/day was the highest mean value, with the maximum contact up to 419 ng/kg body weight/day (47). Wilson and colleagues estimated in a study that the exposure through inhalation for toddlers (1.5–5 years) was 0.23–0.42 ng/kg of body weight per day (52).

Generally, infants (0–6 months old) are the most affected through the alimentary canal all over the population with BPA exposure and its disruptions. The main reason for this exposure is the daily feeding of canned milk formulas in plastic feeder bottles containing PC. Hence, these plastic feeding bottles and canned milk powders make them most vulnerable to BPA’s side effects. Various research studies have been conducted to asses BPA levels and found that the BPA concentration is less than tolerable daily intake (TDI) (47).

Although, BPA exposure through inhalation and dermal routes accounts for less than 5% of all contact sources. The occupational population shares a more significant proportion (50). In a study, 154 composite samples were assessed for BPA analysis and 55 samples with BPA levels ranging from 0.19 to105.0 ng/g, respectively. The experimental results also indicated that canned foods had higher BPA concentrations than other food samples (23).

### Metabolism of bisphenol A

Bisphenol A is highly metabolized and secreted into urine, primarily as a glucuronide conjugate with a half-life of 2 h (53). BPA’s half-life also depends on the glucuronidase enzyme, which activates BPA through deconjugation in the bloodstream, and other organs (54). Furthermore, the valid biomarker of BPA exposure is total BPA (including free or conjugated) urinary concentration (55). The health aspect of free BPA (a weak estrogen) has been primarily observed in animal models. At the same time, limited neonatal human researches are available to check behavioral and executive functional effects, especially during critical child developmental stages and in the shortening of congenital space in male offspring (56, 57). However, several experiments revealed that BPA initially affects hepatic injury (53, 58). After ingestion, most BPA in the liver and gut is rapidly bound with glucuronic acid to release BPA glucuronide (BPA-G) by the glucuronidation process, facilitated by many enzymes (59).

Moreover, being fat-soluble, BPA has high adipose tissue affinity and is then released steadily to other histological structures in humans and mice (60, 61). An investigation to estimate the BPA division in humans highlighted that BPA is demonstrable in almost all human histological structures. In adipose tissues, it ranged from 1.13 to 12.27 ng/g, 0.78 to 3.34 ng/g in the liver, and 1 to 2.35 ng/g in the brain. In breast milk, total BPA was observed as 1.09 ng/mL, out of which 0.41 ng/mL content was identified as unconjugated BPA (6).

Furthermore, the conjugated BPA does not combine with the estrogen receptor (ER); therefore, they are biologically inactive and inert. However, another investigation revealed that BPA-conjugated forms could disturb cellular responsive action throughout membrane ERα contacts, which is responsible for quick signaling feedback (62). In contrast, in trace concentrations, unconjugated BPA (free BPA) can convert into other compounds such as BPA sulfate or BPA-S.

Bio-monitoring records reveal that BPA interaction with humans is prevalent (55, 63). However, there is still massive controversy on the legality of the reported measure of unconjugated BPA in whole blood, plasma, or serum. The discussion point is that adult human blood samples have up to 0.5–2 ng/mL (2.2–8.8 nM) unconjugated BPA. It is very high than the predicted levels of 0.51 μg/kg of body weight per day calculated based on adults’ estimated daily intake (48). Presumably, few of the even most substantial daily consumption records in this range (and lower) depend on the whole day urinary output, with back calculations of 596 German women and men (64).

### Pharmacokinetics of bisphenol A

According to the research conducted by Volkel et al., subjects were given a dose of 54–90 μg/kg of BW/day orally. The results showed that unconjugated BPA was not recognized in urine or serum in any human oral pharmacokinetic (PK) study. Though, the maximum value of finding in the study, 2.27 ng/ml (9.9 nM), is not as much susceptible as more current competencies of 0.05–0.3 ng/ml (0.25–1.76 nM) (65–67).

Another study, utilizing a LOD range of 0.01–0.95 ng/mL, was conducted in which 10 men were given a soup having an unconjugated d6-BPA of 0.097 ng/ml (0.42 nM) at 1.5 h, followed by administration of 29.9 μg/kg of body weight of BPA (68). The d6-BPA was 0.29% of the total BPA, leading researchers to conclude that the sublingual dietary exposure and absorption were reportedly different. Studies regarding the BPA’s pharmacokinetics in rats, mice, and rhesus monkeys using isotope compounds showed that oral consumption of 75 to over 1,000 μg/kg of body weight per day is essential concentrations of unconjugated BPA reported in humans (66, 69–71).

Overall, these changes led to the estimation that unconjugated BPA in the blood is linked with the preparation of the sample, storage, systematic procedure, and exposure conditions. For example, in hospitals where patients may be interacted with BPA from medical equipment or in professional settings (63, 72–78).

### Impact of bisphenol A on health

In animal model studies, BPA has reportedly caused many critical health conditions. Several human-based epidemiological and observational researches showed similar findings on exposure to BPA (47). Disrupted growth susceptibility is higher at certain phases of the life cycle on BPA exposure, halting normal development. Fetal or postnatal development stages are more critical as the body systems are not fully developed. BPA affected growth disruption due to its metabolism and elimination through enzyme systems amalgamation (16, 17). Because of the previous studies, this review will cover the effect of BPA on the human reproductive system, endocrine system, immune system, and carcinogenicity.

#### Endocrine disruption

**FIGURE 2:**
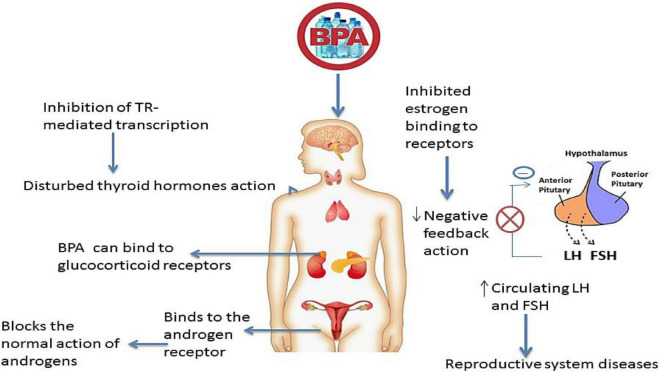
Bisphenol A as an endocrine disruptor. Estrogens negatively affect the release of follicle-stimulating hormone (FSH) and luteinizing hormones (LH). BPA can inhibit the estrogen binding to its receptors at the pituitary level, resulting in high levels of FSH and LH hormones in circulation. This can lead to reproductive system issues such as polycystic ovarian syndrome. BPA acts as anti-androgen by binding with androgen and glucocorticoid receptors and affecting their action. BPA can disturb the action of the thyroid hormone by inhibiting TR-mediated transcription of T_3_-response genes.

The endocrine system is one of the most synchronized and complex systems. BPA is an adverse endocrine-disrupting chemical (EDC) which suppresses or alters hormonal and enzyme synthesis, secretion, release, and transportation. BPA hinders the system’s activity by replacing endogenous hormones with transporter proteins (Figure 2). This alteration changes the free and bound hormonal concentrations present in plasma. This chemical also influences the neuroendocrine function, causing a physiological interruption in the organs. Studies have shown the increased serum level of estradiol in females and reduced testosterone in males due to BPA (79, 80).

Mental health is highly influenced by the disrupter, which causes sex-specific mental impairment and behavioral changes. Disturbed and depressive tendencies rose because “Dehydroepiandrosterone (DHEA),” a neuroactive steroid in males, is decreased, resulting in a possible pathway of the depressive-like phenotype (81). Previous studies regarding the endocrine disruption of BPA are compiled to assess its potent role (Table 2).

**TABLE 2:** Effect of BPA on the endocrine system.

Specimen	Route of exposure	Findings/Health impact	References
Rats	Skin	BPA directly affects the central nervous system on exposure, primarily affecting CA3 pyramidal neurons and GABA_*A*_ receptors.	(124)
Mice	BPA injection (125 mg/kg)	BPA exposure leads to endocrine disruption, which affects the immune system. BPA-induced steroid genesis and Nur77 gene expression in testicular Leydig cells.	(125)
Pregnant female mice	Oral	BPA affects the sex steroid hormone in the urogenital sinus of a fetus. Due to endocrine disruption, BPA increases estrogenic production and adversely affects the fetus’s heart, kidneys, cerebellum, and ovaries.	(126)
Male offspring rats	Injection of BPA (low doses)	Hyperactivity and attention deficit due to endocrine disruption. In the basolateral amygdala, the BPA accumulation results in abnormal synaptic plasticity leading to these defects.	(127)
Rats offspring	Oral (during gestation and lactation)	Metabolic disruption due to raised glutamate and L-α-glutamyl- L-aspartic acid ratio in the hippocampus because the 2 metabolites are involved in the malate-aspartate metabolic shuttle. Myelination, growth, glial, and neuronal development alterations due to endocrine disruption.	(128)

#### Reproductive system

**FIGURE 3:**
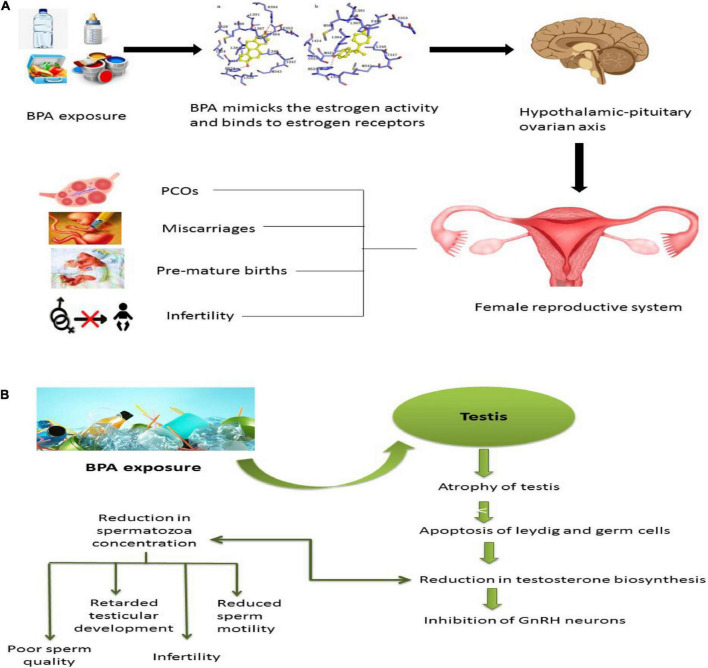
Effect of BPA on the reproductive system. **(A)** When exposed to BPA, females can develop fertility-related issues as it is very similar to estrogen structure and function. It binds to estrogen receptors and causes irreversible alteration to the hypothalamic-pituitary-ovarian axis. BPA will provoke estrogen and thus increase the chances of PCOs, delay puberty, miscarriages, endometriosis, premature births, and most of the time, BPA can cause infertility. **(B)** Exposure of males to bisphenol interferes with the reproductive system. BPA causes atrophy in the testis, apoptosis in Leydig cells and germ cells, and reduction in testosterone biosynthesis, which will either cause the reduction in spermatozoa reduction or inhibition of GnRH neurons. It causes sperm quality and quantity alterations, retardation of testicular development, infertility, and reduction in sperm motility.

Evidential studies have indicated that the reproductive system’s higher interruption susceptibility is observed due to this BPA (Table 3). Being an easy target, the reproductive system undergoes disturbing sex hormone activity and exertion. BPA also distracts the function and primary development of the reproductive system (Figure 3). Recent studies reported the BPA linkage with increased levels of serum luteinizing hormone (LH), estradiol (E2), progesterone, and testosterone (T) while decreased concentrations of serum cortisol (80). A significant association between BPA and higher total testosterone (TT) concentration in serum was also reported (82).

**TABLE 3:** Impact of BPA on the reproductive system.

Specimen	Route of exposure	Findings	References
Female Sprague– Dawley rats	Oral	Significant hormonal disorders altered the structure and functions of the ovaries and uterus.	(129)
KGN ovarian granulosa-like tumor cell line	*In vitro*	Reduction in insulin-like growth factor 1 (IGF-1) induced by FSH and aromatase expression. BPA causes a reduction in granulosa cell DNA synthesis with no changes in DNA fragmentation, showing that BPA does not encourage apoptosis.	(130)
Pregnant women	Oral	Creatinine-identical BPA concentrations caused a reduction in reproducibility. BPA concentration was not altered by the intake of canned fruit, fresh vegetables, fruits, or fresh and frozen fish purchased from the store. High-molecular-weight phthalate and serum tobacco smoke metabolic compound levels were significantly linked with BPA levels.	(131)
Males	Oral	Increased serum total testosterone, prolactin, and estradiol resulted in a reduction in the androgen index.	(132, 133)
Males	Serum	Sexual desire and functionality were decreased in men, followed by premature ejaculation.	(134)
Males	Oral	A higher level of BPA in plasma and seminal plasma has a risk of an increased infertility level.	(135)
Males	Serum administration	The concentration of sperm was decreased, and sperm velocity ratios were increased, followed by a reduction in sperm motility and count.	(133, 136–138)
Females	Oral	The level of Luteinizing hormone and progesterone was increased; hence, the risk for PCOS also increased.	(139, 140)

The endometrial wall thickness and cycle of sex hormones associations are well studied. Scientists observed an age-based relationship between altered endometrial wall thickness and BPA levels (83). Moreover, polycystic ovary syndrome (PCOS) patients exhibited higher BPA serum concentrations than healthy women and patients (84, 85). The researchers also detected BPA’s potential role in PCOS and adverse pregnancy outcomes like premature delivery and miscarriage (86).

Males facing prolonged BPA exposure tend to have low sperm quality, sexual dysfunction, and impaired fertility. The amplitude of lateral head displacement (ALH), Wobble (WOB), Linearity (LIN), Mean Angular Displacement (MAD), sperm concentration, and association with BPA illustrated the fluctuated characteristics and velocity rate reduction. This array results in impaired reproductive function in males (87).

#### Carcinogenicity

**FIGURE 4:**
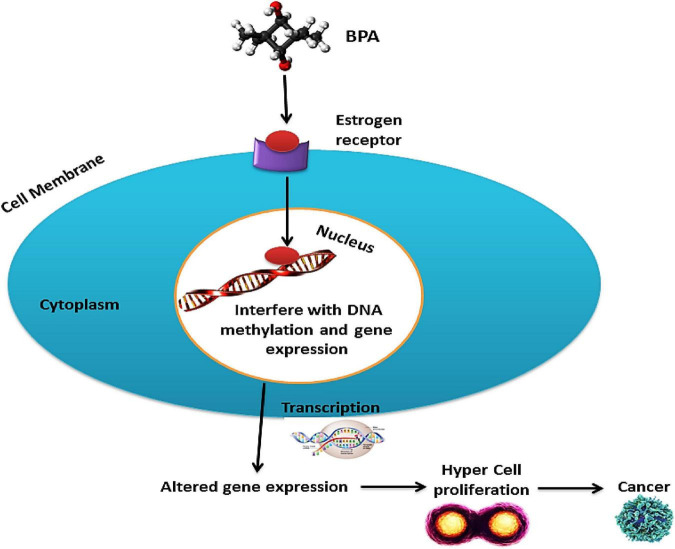
Carcinogenic activity of BPA; BPA interacts with the estrogen receptors and interferes with DNA methylation and gene expression after entering the nucleus. Thus altered gene expression leads to hypercell proliferation, which may lead to cancer.

The incidence of numerous cancer types is rising exceptionally and appears to be linked with BPA (Table 4). It includes breast (88), ovarian, uterus, prostate (89–91), and testicular cancer (92). The findings of the various *in vivo* studies on animals (i.e., mice, rats, etc.) concluded that the raised estrogenic activity depicts the carcinogenic mechanistic action of BPA (93). BPA’s activation of tumorigenesis and cancerous cell development are still under experimentation (88). BPA stimulates cellular responses through binding to ER, although they reflect a weak affinity to each other (Figure 4).

**TABLE 4:** Role of BPA in carcinogenicity.

Specimen	Route of exposure	Findings	References
Rat	Oral	The increased number of Leydig cells and proliferation was caused due to exposure to BPA during the Perinatal period.	(141)
Rat	Antenatal	Ductal carcinoma and ductal hyperplasias were developed due to increased BPA exposure during the perinatal period carcinoma *in situ* and malignant tumors.	(142)
Rat	Neonatal exposure	Polycystic ovary syndrome was reported due to the neonatal exposure to.	(143)
Mouse	Prenatal exposure	Cystadenomas, a high rate of progressive lesions of the oviduct and ovarian cysts, were observed due to Prenatal exposure to BPA.	(144)
Mouse	Neonatal exposure	Increased adenomyosis, cystic endometrial hyperplasia, and leiomyomas developed due to neonatal exposure to BPA.	(144)
Mouse	Oral	BPA exposure was studied in the renal xenograft model, and a high rate of adenocarcinoma of human progenitor cells and prostate intraepithelial neoplasia were reported.	(145)
Mouse	Perinatal exposure	Exposure to BPA during the perinatal period effect the offspring/infant greatly, and it leads to neoplastic lesions and hepatic pre-neoplastic.	(146)
Breeding C57Bl6 mice	Oral	Perinatal contact with BPA amplified the number of TEBs and the progesterone response of the mammary epithelial cells.	(147)
Non-human primates	Oral	BPA exposure to the fetus accelerated mammary epithelial development and a high rate of mammary buds’ density.	(148)

The binding ability of the receptor to hold co-repressors is lost. As the regulation of co-regulators by the BPA–ER complex is disproportionate to the affinity of BPA to ER, the type and expression levels of ER-regulated targets are determinants for the tissue and cellular specificity of the BPA response. BPA can induce genomic responses at concentrations lower than the levels at which it is expected to bind to nuclear ERs (94).

#### Immunosuppressive action

**FIGURE 5:**
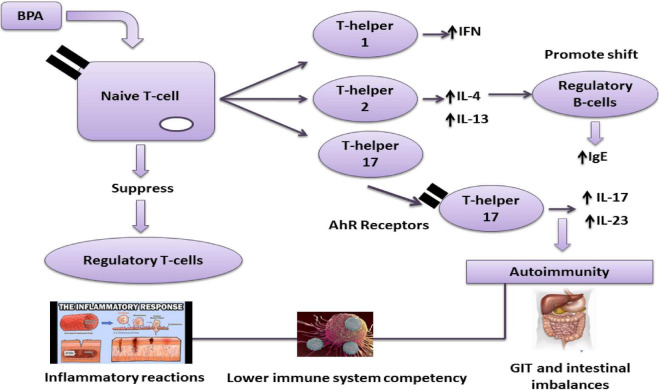
Effect of the BPA immune system BPA can promote autoimmunity *via* T-helpers 1, 2, and 17. Aryl hydrocarbon receptors (AhR) are involved in regulating immune responses, followed by the production of T-helper 17 a critical factor in T-cells in various autoimmune diseases.

Studies have revealed that oxidative stress, immune function, and inflammation are directly related to BPA exposure. The correlation between BPA and the induction of mitochondrial damage and cellular apoptosis resulted in systematic degradation (95–97), causing an alternation in immune cell populations and functioning of the innate and adaptive immune system owing to developmental BPA exposure (Figure 5).

Similarly, it also decreased T regulatory (Treg) cells and up-regulated pro-inflammatory and anti-inflammatory cytokines and chemokines. T1D development in females and males could be accelerated and decline on exposure to BPA (Table 5) (98).

**TABLE 5:** Effect of BPA on the immune system.

Specimen	Route of exposure	Findings	References
Pregnant women	Environmental (inhalation or dermal)	Concentrations of BPA in the mother’s urine were reciprocally associated with odds of increased IL-33/TSLP.	(149)
Humans	Oral and environmental	BPA can harm the immune system’s functions as assessed by CMV antibody levels and allergy or hay fever diagnosis.	(150)
Sprague–Dawley rats	Oral	Ten measurements out of 530 were different from vehicle controls and were primarily associated with dendritic or macrophage cell populations. BPA may have negatively affected the competency of the immune system.	(151)
Mice	Oral	BPA exposure *via* the oral route, in a given amount and for the shown contact period, has minor manipulation of features of the inflammatory response, stimulating immune-mediated diseases of the GIT.	(152)
Mice	Oral	Imbalances induced in intestinal and systemic immune *via* perinatal treatment, the appearance of inflammatory M1 macrophages in gonadal white adipose tissue with signs of aging, combined with a reduction in insulin sensitivity and an enhancement in weight gain.	(153)
Pregnant mice	Oral	Mother exposure to BPA modulated inborn immunity in mature offspring but did not damage the anti-viral adaptive immune response, which is dangerous for virus permission and endurance after influenza virus infection.	(154)
BALB/female mice	Oral	The chances of asthma increased when the mother was exposed to BPA. It might increase the airway hyperresponsiveness in their infants’ lungs as they were bare to BPA before birth and after birth *via* breast milk compared to those exposed to BPA after birth or not treated with BPA at all.	(155)
Adult zebrafish	Oral	Down-regulation in the transcription of genes involved in enzymatic antioxidant defense and impaired anxiety and fear responses.	(156)
Larvae of Labeo rohita	Oral	Oxidative stress and suppressed NF-κB signaling pathway leading to immunosuppression.	(157)
Human granulosa KGN cells	*In vitro*	Damage to biomacromolecules-main targets of oxidative stress was significantly increased after treatment with BPA.	(158)
Adult rats	Oral	BPA-induced systemic oxidative stress change ROS-induced signaling pathways in the brain.	(159)

### Legislations

The primary source of contact for BPA is food for the general population. According to the United States Environmental Protection Agency (EPA), BPA’s reference dose is 50 μg/kg BW/day. EFSA decreased this TDI dose from 50 μg/kg BW/day to 4 μg/kg BW/day in 2015 due to its harmful health effects (12).

A temporary TDI of BPA was set by the CEF panel that was 4 μg/kg BW per day. The board applied an uncertainty factor of 150 for this purpose for different body systems, including the reproductive system, metabolism, neurobehavioral, immune system, and mammary glands. It was done to assess the uncertainty along with inter and intraspecies differences. CEF declared no harmful health impacts by comparing this t-TDI with the estimates of exposure from the diet for any age group and common health concerns from the combined exposure. Therefore, this estimation of exposure to non-dietary sources showed considerable uncertainty compared to dietary sources’ estimates, imploring further research (99).

The rate of exposure from non-dietary sources, including thermal paper or medical devices based on current t-TDI derived by EFSA, was assessed by the SCENIHR or RAC, the Risk Assessment Committee. SCENIHR concluded that neonates in ICU, dialysis patients, and young children with prolonged medical treatment are at higher risk of getting adverse health effects from BPA as it may enter through systemic exposure after exposure to non-oral routes. But besides that, we cannot neglect the benefits of these devices (100). RAC also published and presented a restriction proposal for using BPA in the thermal paper under ECHA as an opinion of BPA’s hazards on human health. The consumers were satisfied by RAC, ensuring the risk of BPA exposure through the thermal paper was controlled. Simultaneously, the chance of getting BPA exposure from cashiers was not declared adequately in control. Severe effects can be faced by pregnant female workers working in a high-exposure environment (101).

#### For infants and children

Higher exposure rates of BPA are reported in Infants and children compared to adults. For breastfed infants, the average (95th percentile) BPA intake was 0.3 μg/kg body weight (BW) per day (1.3 μg/kg BW per day) for the age of 0 and 6 months. At the same time, 2.4 mg/kg BW per day (4.5 mg/kg BW per day) was reported for infants receiving formula from polycarbonate, according to WHO (47). European Union and Brazil have set the permissible limit of 600 μg/kg in infant foods. A study was conducted to assess the limit of BPA in infant formulas and reported the presence of BPA below the required level (0.2–10.2 μg/kg) (102). Another study estimated the BPA intake in different age groups and genders and concluded that their exposure was below the permissible limit (25 μg/kg of body weight/day) of Health Canada. However, dietary exposure to BPA for infants (0.22–0.33 μg/kg of body weight/day) was more than for adults (0.052–0.081 μg/kg of body weight/day). The increased intake was linked to the intake of canned and liquid milk-based infant formula (23).

### Restrictions on the use of bisphenol A

Several restrictions on BPA use have been made after detecting its deteriorating health effects in different countries. The No Observed Adverse Effect Level (NOAEL) of BPA at a dose of 5,000 ng/kg body weight/day through the food intake was considered by the Food and Drug Administration in 2008 in the United States. Some EU Member States’ also banned it in food packaging and containers for children up to 3 years of age, while some have prolonged this ban for other products. Denmark also prohibited using BPA in packaging materials, including cups, or bottles related to food, especially for a breast milk substitute, in 2010. The use of BPA in baby bottles was also banned after 1st March 2011 by EU Commission Directive No. 8/2011 as a preventive measure (103).

Likewise, baby pacifiers containing BPA were also banned in Austria in 2011. All the materials containing BPA that had a chance to contact food were suspended in France by passing a law in 2012 except for industrial equipment, such as tanks and pipes. They also introduced the labeling requirements for food items prepared for children and pregnant women. In 2013, Sweden banned the BPA-containing materials in lacquers of packaging linings for food prepared for children aged 0–3 years (104).

FDA restricted baby bottles and Sippy cups having BPA in 2012 and BPA derivatives in cans on infant formula in 2013 (41, 105, 106). The Commission also conducted an evaluation (started in December 2017) focusing on existing legislation on food packaging; this evaluation ended in 2019. To support the Commission’s evaluation, the Joint Research Centre published a study (107) on the market condition of food packaging not coordinated in the EU. In December 2017, EFSA announced that a strategy was finalized to re-evaluate BPA toxicity with a working group for better results. BPA usage was restricted in thermal paper in January 2020, which would be through registration, evaluation, and authorization. Then restriction of chemicals regulation will decrease the use of BPA in all types of recycling packaging (108).

### Alternatives to bisphenol A

There is a need to find alternative solutions to BPA due to the health hazards of this compound’s use in packaging material. Several studies have been conducted in the previous few years to find the best suitable alternative to BPA having the same properties. Researchers have identified a few compounds that can be used as a replacement for BPA. The low estrogenic and endocrine potential of tetramethyl bisphenol F epoxy resin, bisguaiacol F, and tetramethyl bisphenol F diglycidyl ether is demonstrated in several studies. The researcher also suggested that further research is needed on this group of compounds to assess the potential possible effects of these compounds on human health.

Meanwhile, the authors suggested that they could be viable alternatives to BPA. These BPA substitute-based products are consumed under the label of “BPA-free.” This term gives the impression that the products are safe, but the substitutes’ safety is not fully verified (109, 110).

The increased restrictive rules for using BPA for human health and the environment have become a significant standard for substitution in research and industry (19, 111). Several “bisphenol analogs” have been produced to replace BPA in various applications (112). The most significant market shares are held by BPF (4, 4′-methylene diphenyl), BPS [bis (4-hydroxyphenyl) sulfone], and BPAF [2, 2-bis (4-hydroxyphenyl) hexafluoropropylene] (113–115). Reports and databases are available on the viability of these monomers as BPA substitutes. In Korea, BPS is used for thermal receipt papers and BPF as a water pipe coating agent instead of BPA (116). Although further research is needed to assess these compounds’ potential effects, the authors suggest they could be viable alternatives to BPA (117).

## Conclusion

Bisphenol A can cause multiple organ toxicity after entering the body through the respiratory, dermal, and gastrointestinal tract. It disturbs different cellular mechanisms and hormonal functions by binding with the receptors and activating downstream pathways. The outcomes of BPA exposure are cancers, endocrine disruptions, immunosuppression and reproductive defects. However, there are still ambiguities and many unanswered questions about BPA’s metabolism and its toxic effects. There is a need to elaborate on the combined effects of BPA with other pollutants. Conclusively, BPA-free alternatives should be promoted to avoid these adverse consequences.

### Future recommendations

It is obvious from the current review that more basic non-human primate research and clinical studies are needed to understand the too-complex mechanisms behind BPA activity fully. Nevertheless, several variations across species have been identified, although rats and mice have been demonstrated to be ideal models for investigating the causes of chronic human diseases. Additionally, additional research is needed as a preventative and precautionary measure, especially for developing fetuses and young children, as they are more vulnerable to the harmful effects of this prevalent compound in both developed and developing countries, which requires more attention even through public awareness campaigns. However, because humans are exposed to various pollutants, it is important to consider that BPA may have additive and synergistic effects with other widely used compounds.

## Author contributions

MM, TT, AyS, and SM: conceptualization. AmS, MN, and FT: methodology. TT, FT, and SK: software. MM, TT, FT, and SM: writing—original draft preparation. MM, AyS, and X-AZ: writing—review and editing. X-AZ: supervision. SI: supervision and funding acquisition. All authors have read and agreed to the published version of the manuscript.

## Abbreviations

BW: bodyweight
CAGR: compound annual growth rate
CEF: EFSA panel on food contact materials, enzymes, flavorings, and processing aids
EFSA: European food safety authority
EPA: environmental protection agency
FAO: food and agriculture organization
FDA: food and drug administration
FSH: follicle-stimulating hormone
GABA_*A*_: aminobutyric acid type A
IGF-1: insulin-like growth factor 1
PC: polycarbonate
PK: pharmacokinetic
PVC: polyvinylchloride
RAC: risk assessment committee
SCENIHR: EU scientific committee on emerging and newly identified health risks
TDI: tolerable daily intake
USD: United States dollar
WHO: World Health Organization.
